# A Novel Endovascular Therapy Strategy for Acute Ischemic Stroke Due to Intracranial Atherosclerosis-Related Large Vessel Occlusion: Stent-Pass-Aspiration-resCuE-Micowire-Angioplasty (SPACEMAN) Technique

**DOI:** 10.3389/fneur.2022.798542

**Published:** 2022-02-14

**Authors:** Yingchun Wu, Junmei Wang, Rui Sun, Guanqing Feng, Wenzhao Li, Yuejiang Gui, Yanan Zheng

**Affiliations:** Department of Neurology, ORDOS Central Hospital, Ordos, China

**Keywords:** intracranial atherosclerosis, large vessel occlusion, SPACEMAN, Solumbra, endovascular therapy

## Abstract

**Background:**

There is no clear consensus on the optimal endovascular treatment strategy for patients with ischemic stroke caused by ICAS-related large vessel occlusion (LVO). SPACEMAN, a novel thrombectomy technique that entails passing an aspiration catheter over the stent retriever and then retaining the microwire for angioplasty, has not been described. The aim of this prospective study was to evaluate our initial application of SPACEMAN and compare this technique with the Solumbra technique.

**Methods:**

Forty-four consecutive patients with acute ischemic stroke resulting from ICAS-related LVO were randomly divided into two groups: Solumbra group (*n* = 22) and SPACEMAN group (*n* = 22). Demographic and clinical data were prospectively collected. Modified Rankin Scale (mRS) score of ≤ 2 of anterior circulation and mRS score ≤ 3 of posterior circulation at 3 months post-discharge was regarded as good prognosis.

**Results:**

The SPACEMAN group showed reduced mean time from femoral access to recanalization compared with the Solumbra group (39.55 ± 10.63 min vs. 50.73 ± 9.89 min, *P* = 0.001). The overall recanalization rate in the entire cohort was 93.18% (41/44). At 3-month follow-up, the overall good prognosis rate was 47.73%; 13 patients (59.09%) in the SPACEMAN group and 8 (36.36%) in the Solumbra group showed good prognosis. One patient in the SPACEMAN group (4.55%) and two patients in the Solumbra group (9.09%) developed symptomatic intracranial hemorrhage. The overall mortality rate was 4.55% (2/44).

**Conclusions:**

This study suggests that SPACEMAN exhibits a shorter operation revascularization time than the standard thrombectomy. Complications and prognosis were comparable between the two groups. The safety and efficacy of this novel technique need to be studied in larger patient series.

## Background

Endovascular thrombectomy (ET) is a standard treatment for acute stroke caused by large vessel occlusion (LVO) (1). Atherosclerosis of intracranial arteries is a common cause of stroke in Africa and Asia (2). Intracranial atherosclerosis (ICAS) is the predominant cause of stroke in China (3). However, nearly 30% of stroke patients could not receive reperfusion, which may lead to poor functional outcomes (4). High failure rate of ET in stroke patients is associated with underlying intracranial atherosclerotic disease. Patients with ICAS-related LVO require a higher number of thrombectomy passes and longer procedure time than patients with embolic LVO (5). Moreover, ~1/3 of patients with ICAS-related LVO sustain intraprocedural reocclusion (6). Therefore, development of novel and more efficient endovascular strategies for patients with acute stroke caused by ICAS-related LVO is a key imperative. Rescue therapies such as intraarterial infusion of glycoprotein IIb/IIIa inhibitors, balloon angioplasty, and intracranial stenting are sometimes needed to achieve reperfusion in patients with stroke caused by ICAS-related LVO (5–12). The Solumbra technique is a commonly used strategy for treatment of ischemic stroke caused by ICAS-related LVO; however, there is no clear consensus on the optimal strategy (12). In this study, we present a novel strategy developed at our center for patients with ICAS which we refer to as the “SPACEMAN” technique (stent + pass + aspiration + rescue + micowire + angioplasty). The purpose of this prospective study was to assess the safety and efficacy of SPACEMAN technique for endovascular treatment of stroke patients with ICAS-related LVO.

## Methods

**Figure 1:**
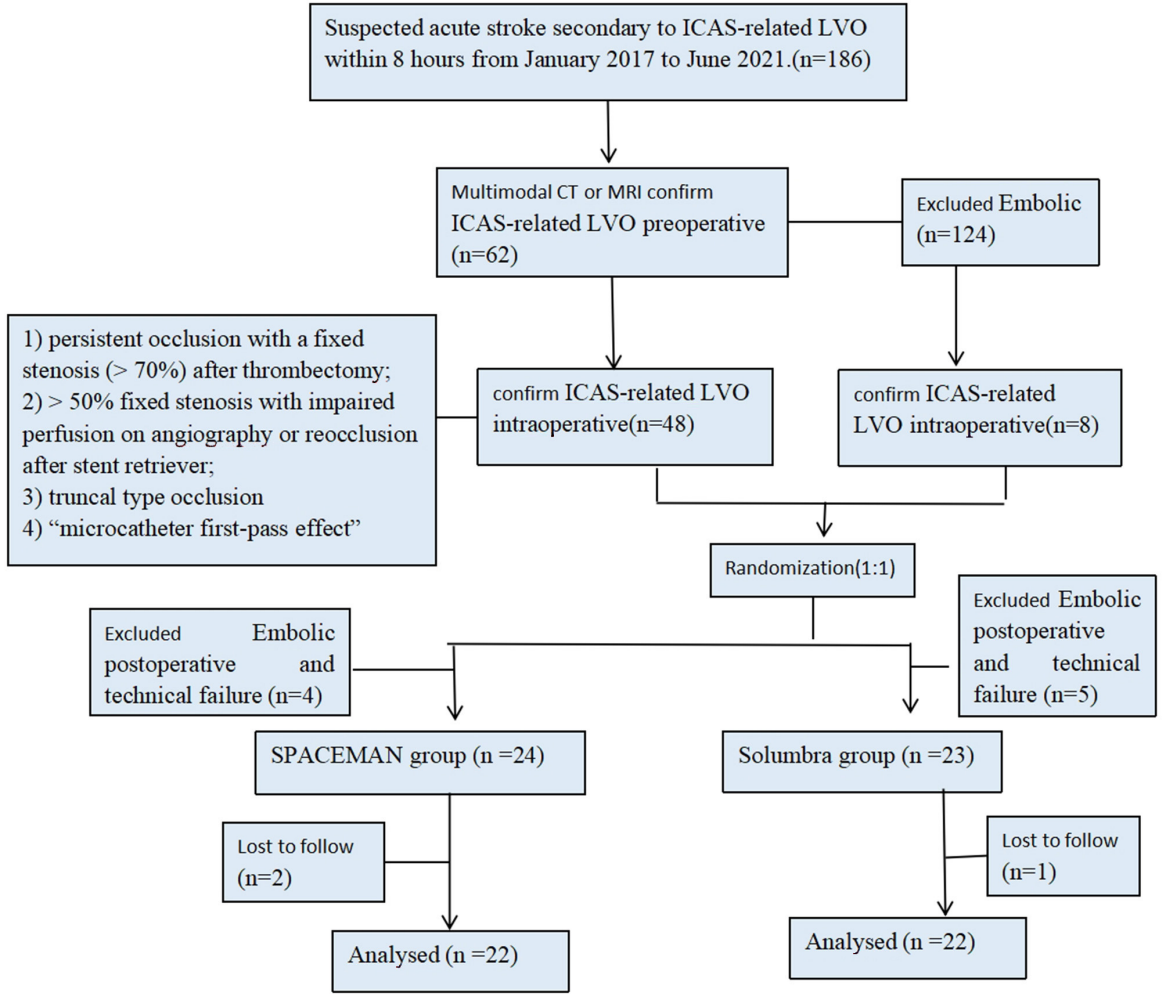
Patient flow diagram.

This prospective study was approved by the institutional ethics committee of the ORDOS Central Hospital. Forty-four consecutive patients with acute stroke who had ICAS-related LVO and were about to undergo endovascular thrombectomy (with either the SPACEMAN technique or the Solumbra technique) at the Department of Neurology of ORDOS Central Hospital were prospectively recruited from January 2017 to June 2021. These patients were assigned to the SPACEMAN group (*n* = 22) or the Solumbra group (*n* = 22) by simple randomization (toss of a coin) (Figure 1). All endpoints were assessed and determined by two experienced specialists in neurology who were blinded to the clinical data.

### Patient Selection

Patients were chosen according to the following inclusion criteria: (1) age ≥18 years; (2) occlusion of the intracranial part of the internal carotid artery, or the first segment of the middle cerebral artery (M1), or intracranial vertebral (V4) and/or basilar artery (BA) occlusion demonstrated by magnetic resonance angiography (MRA), CT angiography (CTA), or digital-subtraction angiography (DSA); (3) period between symptom onset and groin puncture <8 h; (4) modified Rankin scale (mRS) score <3 before the onset of stroke; and (5) National Institute of Health Stroke Scale (NIHSS) score ≥6.

Patients were excluded if they had intracranial hemorrhage identified by computed tomography (CT) or magnetic resonance imaging (MRI) scan within the preceding 3 weeks.

Written informed consent was obtained from all the patients or their immediate relatives.

### Evaluation of ICAS-Related LVO

Patients with ICAS-related LVO were determined as previously described (13–15). The criteria for ICAS-related LVO were (1) persistent occlusion with a fixed stenosis (>70%) after primary thrombectomy; (2) >50% fixed stenosis with impaired perfusion on angiography or reocclusion after stent retriever; and (3) truncal type occlusion or the “microcatheter first-pass effect” according to DSA results. The grade of occlusion was determined based on the Warfarin-Aspirin Symptomatic Intracranial Disease (WASID) criteria (16).

### Imaging Results and Clinical Evaluation

All images were independently examined by two experienced specialists in neurology who were blinded to the clinical data. The following data were collected: baseline demographics, vascular risk factors, NIHSS at admission, intravenous alteplase (tPA) use, time period between symptom onset and groin puncture, procedure time in minutes, thrombectomy techniques, number of attempts, angioplasty use, stenting use, type of stent used, complication rates, and rate of symptomatic hemorrhage. Symptomatic intracranial hemorrhage (sICH) was determined as intracranial hemorrhage, including parenchymal hematoma, subarachnoid hemorrhage or intraventricular hemorrhage, associated with a NIHSS score ≥4 according to the guidelines of the European Cooperative Acute Stroke Study II (ECASSII) (17). Revascularization was determined as modified thrombolysis in cerebral infarction of 2b−3. Follow-up data were collected at 90 days after surgery by two experienced doctors who were blinded to the group identity; good long-term prognosis was defined as mRS score ≤ 2 of anterior circulation and mRS score ≤ 3 of posterior circulation at 90 days.

### Endovascular Therapy and the Solumbar Technique

Endovascular procedure was performed under general anesthesia through the femoral artery. An 8F sheath was placed retrogradely into the femoral artery and diagnostic DSA was performed. After placement of the guiding catheter in the ICA or vertebral artery (VA), aspiration catheter was passed in to the ICA or VA, followed by the SOLUMBAR technique (18, 19). Then, the aspiration catheter was placed to the thrombus 5 min after stent placement, forming manual negative pressure with a 50-mL locked syringe. The thrombus was then retrieved after the aspiration catheter and the stent retriever were removed into the cervical guide catheter.

### SPACEMAN Technique

**Figure 2:**
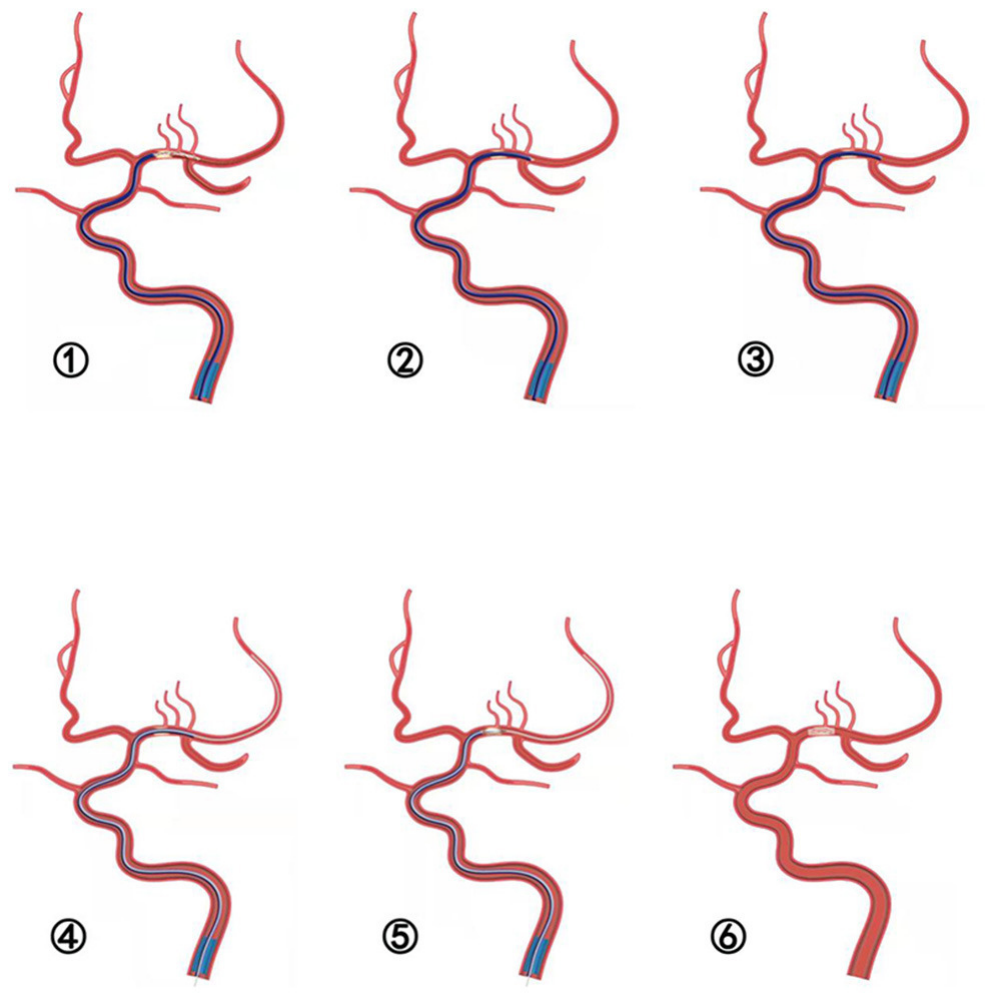
➀-➅ Schematic illustration of the SPACEMAN technique. ➀ The stent is deployed and the aspiration catheter is placed to the thrombus. ➁ The aspiration catheter is placed through the thrombus over the distal tip of stent retriever. ➂ The stent retriever is removed under negative suction without changing the position of the aspiration catheter. ➃ A 300 cm microwire is steered through the occlusion lesion to the distal segment under the guidance of the aspiration catheter. ➅ Angiography performed after the aspiration catheter is withdrawn proximal to the occlusion. ➆ Angioplasty of balloon or stent is performed.

The novel technique referred to as SPACEMAN is first described in this study. In brief, after deployment of the guiding catheter into the ICA or VA, a microwire and microcatheter were navigated into the occluded vessel which was distal to the thrombus. At 5 min after stent placement (Figures 2➀,➁), the aspiration catheter was placed through the thrombus over the stent retriever forming manual negative pressure with a 50-mL locked syringe. Once the aspiration catheter was at the end of the stent retriever (Figure 2➁), the stent retriever was withdrawn under continuous negative pressure without changing the position of the aspiration catheter (Figure 2➂). When the blood flow was maintained, over 20 mL of blood was withdrawn to ensure that the withdrawn blood did not contain thrombus. Then, a 300 cm microwire was passed through the occlusion to the end segment under the guidance of the aspiration catheter (Figure 2➃). Angiography was performed after the aspiration catheter was withdrawn proximal to the occlusion (Figure 2➄). The degree of occlusion was evaluated by angiography and cerebral hemorrhage was excluded by CT. Then the balloon or stent was advanced to perform angioplasty (Figure 2➅).

**Figure 3:**
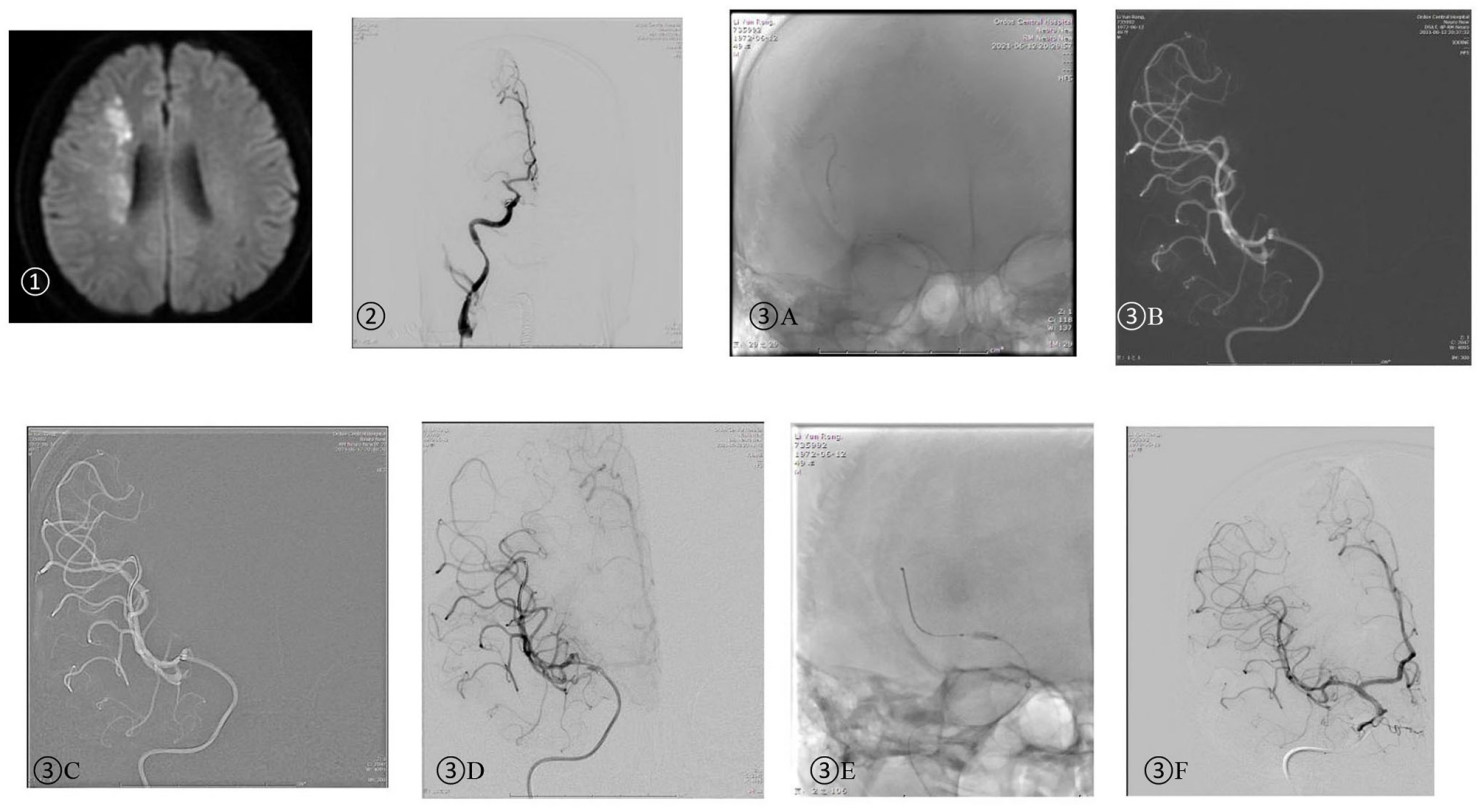
A 49-year-old man was admitted due to left limb numbness 12 h ago and left limb paralysis 6 h ago. NIHSS: 10.➀ Head DWI: watershed infarction of the right hemisphere; ➁ DSA: right MCA M1 initial part occlusion; ➂ SPACEMAN technique: **(A)** The aspiration catheter was placed near the occlusion segment and the stent retriever was released after verification of true vascular cavity by the microwire. **(B)** The aspiration catheter was placed through the thrombus over the stent retriever forming manual negative pressure. Once the aspiration catheter reached the end of the stent retriever (Figure 2➁), the stent retriever was then withdrawn under continuous negative pressure. **(C)** A 300 cm microwire was placed at MCA M2 distal segment under the guidance of the aspiration catheter. **(D)** Angiography was performed after the aspiration catheter was withdrawn proximal to the occlusion and showed severe M1 stenosis. **(E)** The balloon-expandable stent was delivered to the stenotic segment under the guidance of the aspiration catheter. **(F)** DSA performed after angioplasty showed complete recanalization.

This procedure was performed as per the following steps: (1) stent placement; (2) passage of aspiration catheter over the stent; (3) negative aspiration; (4) microwire; (5) angioplasty; and (6) revascularization. Therefore, this technique adopted by us is named as the SPACEMAN technique (Figure 3).

### Statistical Analysis

Kolmogorov-Smirnov normality test was used to assess normality of continuous variables. Quantitative variables exhibiting Gaussian distribution were expressed as mean ± standard deviation (SD) and between-group differences were assessed using the Student *t*-test. Non-normally distributed continuous variables were presented as median (25th percentile, 75th percentile) [M (P_25_, P_75_)] and the between-group differences were assessed using the Wilcoxon rank-sum test. Categorical variables were described as frequency (%) and between-group differences were assessed using the Chi-squared test. Ordinal variables were also expressed as frequency (%) and between-group differences were assessed using the Chi-squared test. *P*-values < 0.05 were considered indicative of statistical significance.

## Results

Forty-four patients with acute ischemic stroke were treated by thrombectomy at our hospital (32 male and 12 female; mean age: 58.93 ± 9.31 years). The mean NIHSS score was 15.89 ± 4.55. Baseline characteristics are summarized in Table 1. No significant between-group difference was observed with respect to age, sex, vessel risk (hypertension, diabetes mellitus, hyperlipidemia, atrial fibrillation, coronary artery disease, ischemic stroke and smoking), NIHSS on admission, DWI-ASPECTS score, intravenous thrombolysis or vessel occlusions in general. The time from symptom onset to puncture, rescue endovascular therapy (balloon angioplasty and/or stent placement) for ICAS and the rate of embolization were also similar between the two groups. No technique-related complications or embolization occurred in the SPACEMAN group. The average time from femoral access to recanalization in the SPACEMAN group was shorter than that in the Solumbra group (39.55 ± 10.63 vs. 50.73 ± 9.89 min, *P* = 0.001).

**Table 1:** Baseline demographic and clinical characteristics of the study population.

**Variables**	**Total**	**SPACEMAN**	**Solumbra**	***t*/χ^2^*/z***	***P-*value**
	**(*n* = 44)**	**(*n* = 22)**	**(*n* = 22)**		
Age, (mean ± SD)	58.93 ± 9.31	58.77 ± 9.53	59.09 ± 9.30	−0.112	0.911
Male, *n* (%)	32 (72.73%)	16 (72.73%)	16 (72.73%)	0.000	1.000
Hypertension, *n* (%)	25 (56.82%)	13 (59.09%)	12 (54.55%)	0.093	0.761
Diabetes mellitus, *n* (%)	9 (20.45%)	4 (18.18%)	5 (22.73%)	0.140	0.709
Hyperlipidemia, *n* (%)	13 (29.55%)	6 (27.27%)	7 (31.82%)	0.109	0.741
Atrial fibrillation, *n* (%)	1 (2.27%)	1 (4.55%)	0 (0.00%)	–	1.000
Coronary artery disease, *n* (%)	9 (20.45%)	5 (22.73%)	4 (18.18%)	0.000	1.000
Ischemic stroke, *n* (%)	11 (25.00%)	5 (22.73%)	6 (27.27%)	0.121	0.728
Smoking, *n* (%)	25 (56.82%)	13 (59.09%)	12 (54.55%)	0.093	0.761
Baseline NIHSS score (mean ± SD)	15.89 ± 4.55	16.05 ± 5.10	15.73 ± 4.04	0.229	0.820
DWI-ASPECTS score, *M* (P_25_, P_75_)	7.00 (6.00, 8.00)	7.00 (6.00, 8.00)	7.00 (6.00, 8.00)	−0.380	0.704
Intravenous thrombolysis, *n* (%)	21 (47.73%)	10 (45.45%)	11 (50.00%)	0.091	0.763
Occluded site, *n* (%)				0.484	1.000
Internal carotid artery	9 (20.45%)	4 (18.18%)	5 (22.73%)		
Middle cerebral artery	24 (54.55%)	12 (54.44%)	12 (54.55%)		
Basilar artery	7 (15.91%)	4 (18.18%)	3 (13.64%)		
Intracranial vertebral artery	4 (9.09%)	2 (9.09%)	2 (9.09%)		
TOP (min), *M* (P_25_, P_75_)	305 (270,370)	300 (270,370)	315 (270,370)	−0.247	0.805
TO, mean ± SD	45.14 ± 11.62	39.55 ± 10.63	50.73 ± 9.89	−3.613	0.001^*^
Embolization, *n* (%)	1 (2.27%)	0 (0.00%)	1 (4.55%)	–	1.000

*DWI, diffusion weighted imaging; ASPECTS, alberta stroke program early CT score; TOP, time from onset to puncture; TO, time of operation (from puncture to recanalization)*.

* *Means statistical significance*.

The clinical outcomes were similar in the two groups (Table 2). Forty-one patients achieved reperfusion (recanalization rate: 93.18%). In the SPACEMAN group, successful reperfusion (TICI ≥2b) was achieved in 95.45% of cases, as opposed to 90.91% in the Solumbra group (*P* = 1.000). Two patients (9.09%) in the Solumbra group and one patient (4.55%) in the SPACEMAN group sustained sICH. The number of patients who had overall mRS score ≤ 2 at 90 days was 13 (59.09%) in the SPACEMAN group and 8 (36.36%) in the Solumbra group. Twelve patients (54.55%) with anterior circulation lesions achieved favorable outcomes (mRS: 0–2) in the SPACEMAN group and 8 patients (36.36%) in the Solumbra group. Four patients (18.18%) with posterior circulation lesion achieved favorable outcomes (mRS: 0–3) in the SPACEMAN group and two patients (9.09%) in the Solumbra group. Both patients with anterior circulation lesions and those with posterior circulation lesions showed a trend to better results in the SPACEMAN group, although the between-group difference was not statistically significant.

**Table 2:** Comparison of primary and secondary outcomes.

	**Total**	**SPACEMAN**	**Solumbra**	**χ^2^**	***P-value***
	**(*n* = 44)**	**(*n* = 22)**	**(*n* = 22)**		
TICI grade ≥2b recanalization, *n* (%)	41 (93.18%)	21 (95.45%)	20 (90.91%)	0.000	1.000
Symptomatic intracranial hemorrhage, *n* (%)	3 (6.82%)	1 (4.55%)	2 (9.09%)	0.000	1.000
Overall mRS ≤ 2 at 90 days, *n* (%)	21 (47.73%)	13 (59.09%)	8 (36.36%)	2.277	0.131
Anterior circulation mRS ≤ 2 at 90 days, *n* (%)	20 (45.45%)	12 (54.55%)	8 (36.36%)	1.467	0.226
Posterior circulation mRS ≤ 3 at 90 days, *n* (%)	6 (13.64%)	4 (18.18%)	2 (9.09%)	0.193	0.660
Mortality at 90 days, *n* (%)	2 (4.55%)	1 (4.55%)	1 (4.55%)	0.000	1.000
**Rescue treatments**, ***n*** **(%)**					
Balloon angioplasty, *n* (%)	24 (54.55%)	11 (50.00%)	13 (59.09%)	0.367	0.545
Stent placement, *n* (%)	17 (38.64%)	7 (31.82%)	10 (45.45%)	0.863	0.353

## Discussion

Asian countries have a high prevalence of ICAS, and the highest incidence has been reported in China (20). Arterial occlusion caused by ICAS may be an important reason of stent retriever thrombectomy failure, especially in Asian patients (21, 22). Therefore, there is a need to develop novel strategies for endovascular treatment in acute stroke patients with underlying ICAS.

To the best of our knowledge, this is the first prospective study focusing on the technique of endovascular treatment for ischemic stroke with intracranial atherosclerosis-related LVO. The SPACEMAN technique described in this work is a novel thrombectomy technique that has been pioneered at our center. In this study, we compared the efficacy and safety of the SPACEMAN and the Solumbra techniques in the treatment of ICAS-related LVO, and we found that the SPACEMAN technique was associated with reduced time to achieve recanalization compared with the Solumbra technique; however, there were no significant between-group differences with respect to symptomatic intracranial hemorrhage, mRS score, or mortality at 90-day follow-up after discharge.

In the SPACEMAN group, successful reperfusion (TICI 2b and 3) was achieved in 95.45% of cases, as opposed to 90.91% in the Solumbra group, which was higher than that in previous reports (5), and showed a trend to higher recanalization rate in the SPACEMAN group, although lacking statistical significance.

The Solitaire FR stent retriever and the Penumbra aspiration catheter have been used in the Solumbra technique for mechanical thrombectomy (18, 19). Thrombectomy performed using the Solumbra technique shows good results as it combines mechanical thrombectomy with both stent retriever and a thrombus aspiration. However, patients with ICAS-related LVO seem to be refractory to thrombectomy, leading to a low recanalization rate and increased rate of re-occlusion and arterial dissection.

The advantages of the SPACEMAN technique are described as follows. First, in this technique, the aspiration catheter is passed through the proximal thrombus over the stent retriever and enters the distal vessel with continuous negative suction. The time spent on stent retrieval due to re-occlusion or arterial dissection can be saved because clot retrieval is performed first. Second, a 300 cm microwire is placed distal to the occlusion lesion through the aspiration catheter and angiography is performed after the aspiration catheter is withdrawn proximal to the occlusion; this facilitates quick rescue therapy after full evaluation of the lesion. The rescue strategy entails detaching the stent retriever or allowing balloon expansion in patients meeting certain criteria. SPACEMAN technique is different from other techniques entailing combined use of aspiration and the stent retriever, such as Solumbra, CAPTIVE, and ARTS, in that the SPACEMAN technique entails steering the aspiration catheter through the occlusion lesion (18, 19, 23, 24). Theoretically, steering the aspiration catheter through the lesion may cut the thrombus, leading to distal embolization. However, no embolization event was observed in the SPACEMAN group in the present study. Another concern is that the catheter may disturb the occlusion lesion and cause partial arterial dissection. In the present study, the aspiration catheter was passed through the occlusion lesion uneven fully using the stent anchor technique and no arterial dissection occurred; this aspect is similar to the ADVANCE technique in that the thrombectomy is performed only once during operation in both techniques (25). The difference between SPACEMAN and ADVANCE is that the 300 cm microwire is preserved after thrombectomy in the former, allowing for rescue therapy. Third, placement of balloon or stent is easier under the guidance of the aspiration catheter, minimizing vascular damage, especially with use of balloon-expandable stent. The relatively stiff delivery platform may limit the application of balloon-mounted stents in intracranial lesions due to the challenge of navigating around the carotid siphon. However, the relevance of this limitation has declined as the currently available aspiration catheters can be easily navigated through the siphon (26). Fourth, re-occlusion can occur after thrombectomy in ICAS patients, which may necessitate repeat thrombectomy or rescue therapy. All patients in our study underwent thrombectomy only once, which may be due to precise pre- and intra-operative diagnosis of ICAS-related LVO (13–15).

Stent retriever devices and aspiration catheters are useful for removing thrombus, but are not suitable for treating the underlying ICAS. Rescue therapy, such as balloon angioplasty and intracranial stenting, has been described as a rescue procedure in cases where thrombectomy fails (27–30). Rescue therapy may be an acceptable option since it maintains vascular patency in cases with persistent occlusion or a higher likelihood of poor prognosis; however, application may necessitate the administration of antiplatelet drugs or angioplasty that can increase the risk of bleeding. In the current study, the rate of balloon dilatation and stent placement were 54.55 and 38.64%, respectively. The incidence of sICH in our entire cohort was only 6.82%; the incidence in the SPACEMAN group was 4.55%, which is consistent with the reports of HERMES (4%) and EAST (4.3 %) (31, 32). The reasons may be associated with reduced vascular endothelial cell injury, since the aspiration catheter can expand the partial plaque during passage through the occlusion lesion and less thrombectomy is needed.

The good prognosis rate in our study was 47.73% at 90-day follow-up. Both patients with anterior circulation lesions and those with posterior circulation lesions showed a trend to better results in the SPACEMAN group, although the between-group difference in this respect was not statistically significant.

As for the prognosis of endovascular treatment for ICAS-related LVO in other studies, Kim et al. (33) reported a low rate of good prognosis (36.3% with mRS score 0–3) at 3 months after endovascular therapy in 19 patients with ICAS-related acute vertebrobasilar occlusion. The main reasons for the poor prognosis after endovascular treatment in Kim's study may be heterogeneity of the rescue therapy after primary thrombectomy, the low rate of angioplasty/stent placement, and long procedure time. However, Lee et al. (34) reported a high rate of good outcome (60% with mRS score 0–2) at 3 months after endovascular treatment in patients with ICAS-related acute vertebrobasilar occlusion (*n* = 15). In Lee's study, the NIHSS scores were low and the procedure time was short, which may explain the good prognosis after endovascular therapy.

Some limitations of our study should be acknowledged. The number of patients with acute ischemic stroke in our study was small, which was not suitable for subgroup analysis, especially for anterior circulation LVO. In addition, the follow-up time was not long enough for patients undergoing stenting. The long-term benefits and risks of SPACEMAN technique for treatment of ICAS-related LVO remain to be elucidated in the future.

## Conclusions

Our study suggests that SPACEMAN is a novel, safe, and effective endovascular treatment technique that combines aspiration catheter, stent retriever, and rescue therapy in patients with acute ischemic stroke caused by ICAS-related LVO. However, larger studies are required to provide more definitive evidence.

## Data Availability Statement

The original contributions presented in the study are included in the article/supplementary material, further inquiries can be directed to the corresponding author/s.

## Ethics Statement

The studies involving human participants were reviewed and approved by ORDOS Central Hospital. The patients/participants provided their written informed consent to participate in this study. Written informed consent was obtained from the individual(s) for the publication of any potentially identifiable images or data included in this article.

## Author Contributions

Material preparation, data collection and analysis were performed by YW. The first draft of the manuscript was written by YW, JW, RS, GF, WL, YG, and YZ commented on previous versions of the manuscript. All authors contributed to the study conception, design, read and approved the final manuscript.

## Funding

ORDOS Industrial Innovation Talent Team Award.

## Conflict of Interest

The authors declare that the research was conducted in the absence of any commercial or financial relationships that could be construed as a potential conflict of interest.

## Publisher's Note

All claims expressed in this article are solely those of the authors and do not necessarily represent those of their affiliated organizations, or those of the publisher, the editors and the reviewers. Any product that may be evaluated in this article, or claim that may be made by its manufacturer, is not guaranteed or endorsed by the publisher.
